# Attention Deficit Hyperactivity Disorder (ADHD) among Children Aged 6 to 17 Years Old Living in Girja District, Rural Ethiopia

**DOI:** 10.1155/2019/1753580

**Published:** 2019-04-14

**Authors:** Hirbaye Mokona Lola, Habte Belete, Abebaw Gebeyehu, Aemro Zerihun, Solomon Yimer, Kassech Leta

**Affiliations:** ^1^Department of Psychiatry, College of Medicine and Health Sciences, Dilla University, Dilla, Ethiopia; ^2^Department of Psychiatry, College of Medicine and Health Sciences, Bahir Dar University, Bahir Dar, Ethiopia; ^3^Institute of Public Health, College of Medicine and Health Sciences, University of Gondar, Ethiopia; ^4^Department of Clinical Psychiatry, Amanuel Mental Specialized Hospital, Addis Ababa, Ethiopia; ^5^Debre Birhan Health Science College, Debre Birhan, Amhara Region, Ethiopia

## Abstract

**Objective:**

Attention deficit hyperactivity disorder (ADHD) is one of the most common behavioral disorders in childhood with long-term outcomes. Although ADHD is the most studied behavioral disorders of childhood in developed countries, few studies have been conducted in Ethiopia. The aim of this study was to determine the prevalence of ADHD in rural parts of Ethiopia.

**Method:**

A cross-sectional study was conducted from May to June 2015 among children aged 6 to 17 years living in rural areas. A multistage cluster sampling technique was used to select 1302 participants. The Disruptive Behavior Disorder Rating Scale was used to collect the data. Logistic regression analysis was used to see statistically significant variables.

**Result:**

The prevalence rate of attention deficit hyperactivity disorder (ADHD) among children was 7.3%. Being male (Adjusted Odds Ratio (AOR) = 1.81, 95% CI: (1.13, 2.91)); living with a single parent (AOR = 5.0, 95% CI: (2.35, 10.65)); child birth order/rank (AOR = 2.35, 95% CI: (1.30, 4.25)); and low family socioeconomic status (AOR = 2.43, 95% CI: (1.29, 4.59)) were significantly associated with ADHD.

**Conclusion:**

The ADHD prevalence rate was found to be similar with global reports. Prevention and early management of maternal complications is important to reduce the prevalence of ADHD among children.

## 1. Introduction

Attention deficit hyperactivity disorder (ADHD) is one of the most common neurodevelopmental disorders with childhood onset [1]. ADHD is a severe developmental disorder characterized by pervasive and impairing symptoms of inattention, hyperactivity, and impulsivity that occur before the age of seven. The behavioral disturbance of children with ADHD significantly impairs their social, academic, or occupational functioning. ADHD has three subtypes which includes the inattentive subtype, the hyperactive/impulsive subtype, and the combined subtype [2].

The worldwide pooled prevalence of ADHD for children aged 18 years and below was 7.2% from the systematic review and meta-analysis of 175 studies worldwide [3]. From 86 review and meta-analysis studies, the prevalence of ADHD ranges from 5.9 to 7.1% in children and 5% in adults [4]. In a recent report from the Centers for Disease Control and Prevention, the prevalence rate of ADHD was 6.8% in US children aged 3-17 years and has increased by 21.8% between 2003 and 2007 [5]. From a national survey among US children aged 4 to 17 years, the prevalence rate of ADHD was 11% and this has increased by 42% from 2003 to 2011 [6]. In other studies, the prevalence rate of ADHD has been reported to be 8.2% among children aged 6 to 17 years in the USA [7], 6.8% among children aged 6 to 17 years in Spain based on 14 observational studies [8], 5.8% among children aged 6 to 17 years in Brazil using the Diagnostic and Statistical Manual of Mental Disorder Fourth Edition (DSM-IV) and 1.5% using the 10th revision of the International Statistical Classification of Diseases and Related Health Problems (ICD-10) [9], and 10.03% among children aged 4 to 12 years in Venezuela [10]. In Saudi Arabia, the prevalence rate of ADHD in children aged 6 to 13 years has been reported to be 16.4% for the combined subtype (ADHD-C), 16.3% for the inattention subtype (ADHD-I), and 12.4% for the hyperactive/impulsive subtype (ADHD-HI) [11]. Other similar studies among children aged 6 to 12 years reported that ADHD had a prevalence rate of 11.6% (of which 6.3% belonged to the inattention subtype, 2.2% belonged to the hyperactive/impulsive subtype, and 3.1% belonged to the combined ADHD subtype) [12]. In Iranian children aged 3 to 6 years, ADHD had a prevalence rate of 25.8% based on parents' evaluation and 17% based on their teacher's evaluation [13].

Studies in Africa are limited, but ADHD was reported in certain studies. In Africa, the prevalence of ADHD ranges between 5.4% and 8.7% among school children and 1.5% in the general population. However, it ranges from 45.5% to 100.0% among special populations of children with possible organic brain injury [14]. The prevalence of ADHD was reported to be 6.9% in Egypt [15], 3.2 to 23.15% in Nigeria [16, 17], 6.3% in Kenya [18], 6% in Congo [19], 19.7% in Egypt [20], and 1.5% in Ethiopia among children aged 5 to 14 years [21].

ADHD is a result of complex dealings between genetic, environmental, and developmental traits, and genetic factors are credited for determining about 80% of the cases [22]. The main factors mentioned to have an association with ADHD across studies were diagnostic criteria, source of information, and origin of the studies [23]. Other demographic variables like parents' low education, mother's occupation, and low socioeconomic status [11], as well as gender (male gender), child rank (birth order), mother's education level, and living with a single parent [13, 20, 24] were all risk factors of ADHD. Family-related factors like mother's smoking and drinking behavior, nonvaginal delivery, and late starting of school discussed in [25]; watching television/playing video games, participation in sports, and two-parent family structure discussed in [26]; and parental psychiatric disorders, previous abortion, unwanted pregnancy, history of trauma, cesarean delivery, substance use during pregnancy, head trauma, and epilepsy discussed in [27] all contributed to the risk of ADHD.

Children with ADHD are at risk of dropping out of school, becoming pregnant as a teenager, and committing criminal behavior. Untreated ADHD increases the risk for future complications such as poor academic performance and learning delay, low self-esteem, poor social skills, and increased susceptibility to physical injury in childhood [28]. ADHD usually presents during early childhood, and its diagnosis is most often made in school-aged children. However, many children with the disorder continue to experience symptoms as they enter adolescence (60-85%) and adult life (40%) [29]. Children with ADHD are at risk of a wide range of psychiatric disorders [30]. Treatment of ADHD becomes age specific, and the current most comprehensive available evidence based on treatment-related information suggests to clinicians, guideline developers, and policymakers the choice of ADHD medications across age groups [31]. Thus, age-specific information about the magnitude of ADHD is very important in low-income countries to frame appropriate treatment guidelines. Even though there are many studies conducted on the prevalence and risk factors of childhood ADHD in developed countries, limited studies have been conducted in East African, especially in Ethiopia. Therefore, this study will estimate the prevalence and associated factors of ADHD among children aged 6-17 years old living in rural Ethiopia.

## 2. Methods

### 2.1. Study Area

The study was conducted in the Girja district, which is found in the Guji zone, Southern Oromia regional state, Ethiopia. It is located in the southern part of Ethiopia, which is 559 kilometers from Addis Ababa. It has a total population of 62,083 (31,289 males and 30,793 females), 12,934 households, 19,357 children with ages from 5 to 15 years, and 20 kebeles (the smallest administrative unit).

### 2.2. Study Design and Population

A study using a community-based cross-sectional design was conducted among children aged 6 to 17 years old living in rural areas of the Girja district, Guji zone, Oromia regional state, Ethiopia, from May 09 to June 02, 2015. Study populations were randomly selected and children aged 6 to 17 years who lived in the study area for at least six months prior to the study were targeted. All children aged 6 to 17 years old living in the rural area of the Girja district in the selected kebeles (the smallest administrative unit) were selected as the study population. Children aged 6 to 17 years old who had informants present during the study period and children with a permanent residence in the Girja district were included. Children without informants were not included. We included 1302 children for the study.

A multistage cluster sampling technique was employed. The twenty kebeles (the smallest administrative unit) in the district were classified as a cluster. Out of the twenty kebeles, five kebeles were selected by simple random sampling. A total of 5351 children aged 5 to 15 years old were living within 3573 households in the five selected kebeles. From each kebele, two Gotts (the smallest subunits of kebeles/villages) were selected randomly. Then, we selected children aged 6 to 17 years old from each household which had an eligible child. When more than one eligible child was available in the household, the lottery method was used to select one child.

### 2.3. Data Collection Method and Instruments

The presence of ADHD among children aged 6 to 17 years old was assessed by using the Disruptive Behavior Disorder Rating Scale based on the DSM-IV criteria [32]. The scale consists of 45 items representing symptoms of disruptive behavior, and out of the 45 items, 18 items are used for the assessment criteria of ADHD. The Disruptive Behavior Disorder Rating Scale is a proxy-administered (parent or teacher) questionnaire that is based on the Diagnostic and Statistical Manual of Mental Disorders, 4th Edition (DSM-IV) diagnostic criteria for attention deficit hyperactivity disorder (ADHD). It has subscales for the inattention, hyperactivity-impulsivity, and combined subtypes. The Disruptive Behavior Disorder Rating Scale has 45 items, but we used only 18 items for assessing ADHD in this study. Each symptom was rated on a 4-point scale indicating the occurrence and the severity of symptoms: 0 (not at all), 1 (just a little), 2 (pretty much), or 3 (very much). Inattention requires six or more counted behaviors from questions 1 to 9 as an indication of the predominantly inattentive subtype. Hyperactivity-impulsive requires six or more counted behaviors from questions 10 to 18 on impulsivity as an indication of the predominantly hyperactive/impulsive subtype. Combined requires six or more counted behaviors each from both the subtype inattention and subtype hyperactivity/impulsivity dimensions. Children who had acquired scores of six or more on these items were consider as having ADHD based on the original scoring [33]. The Disruptive Behavior Disorder Rating Scale was pretested for validity in our setup and was found to be easily understood by the participants with internal consistency (Cronbach's alpha = 0.8). Data was collected from parents or caretakers using the face-to-face interview technique using the Disruptive Behavior Disorder Rating Scale items that were translated to the local language (Afan Oromo). Maternal health status during pregnancy was assessed by whether the mother was sick as severe as hospitalized during pregnancy. A semistructured questionnaire was used; it was translated to the local language (Afan Oromo) by experts in both languages and translated back to English by another person to ensure consistency and accuracy. Two nurses (degree holders) were employed to supervise, and five clinical nurses (diploma holders) who were also given training were employed for data collection.

### 2.4. Data Analysis

First, the data was checked for completeness and consistency. Then, it was coded, entered, and cleaned before and during data processing by using EpiData version 3.1 and exported to Statistical Package for the Social Sciences version 20 for analysis. Association between the dependent and independent variables was assessed using logistic regression. Variables with a *P* value less than 0.2 during bivariate analysis were entered into multivariable logistic regression. The strength of the association was presented by crude odds ratio and adjusted odds ratio with their 95% confidence interval (CI). Variables that have *P* values less than 0.05 were considered statistically significant.

### 2.5. Ethical Consideration

Ethical clearance was obtained from the Ethical Review Committee of the University of Gondar and formal permission letters were taken from the Girja district administration and health offices. Written assent was taken from the parents/guardians and informed consent was taken from the mothers/guardians for their voluntary participation. Confidentiality was maintained by omitting personal identification. Children with ADHD were referred to the psychiatric clinic.

## 3. Results

### 3.1. Sociodemographic Characteristics of Children Aged 6 to 17 Years

From the 1302 proposed participants, 1238 children were involved with a response rate of 95.1%. There was a total of 640 males (51.7%), and 894 (72.2%) of the children were between the ages of 6 and 12 years. Majority of the children, 1208 (97.6%), were Oromo by ethnicity and 1003 (81%) were Protestant by religion (Table 1).

### 3.2. Sociodemographic Characteristics of Family

Majority of the parents, 1202 (97.1%), were married and 36 (2.9%) were divorced/widowed. From the total number of mothers, 716 (58.5%) could not read and write. Regarding family size, 68.3% (845) of the families had greater than four children in the house. Of the children, 685 (55.3%) were the first child in birth order/rank (Table 2).

Other clinical- and maternal-related factors are listed in Table 3.

### 3.3. Prevalence of ADHD among Children Aged 6 to 17 Years

The prevalence of ADHD was 7.3% (90) (95% CI: 5.8%, 8.8%). From this, 57 (63.3%) were from the ADHD-IN subtype, 22 (24.5%) were from the ADHD-HI subtype, and 11 (12.2%) were from the ADHD-C subtype. ADHD subtypes clustered by sex are shown in Table 4.

### 3.4. Factors Associated with ADHD among Children Aged 6 to 17 Years

During bivariate analyses of ADHD, sex, family marital status, living circumstances of the child, childbirth order or rank, family size, socioeconomic status of the family (by wealth index), and maternal health status during pregnancy fulfilled the minimum need (*P* value at 0.2 significance). Those independent factors were entered into multivariate regression for further analysis. During the multivariate analysis of all the explanatory variables of ADHD, it was found that being male (AOR = 1.81, 95% CI: (1.13, 2.91)), child living circumstance (living with single parent) (AOR = 5.00, 95% CI: (2.35, 10.65)), child birth order/rank (AOR = 2.40, 95% CI: (1.30, 4.25)), and family socioeconomic status (AOR = 2.43, 95% CI: 1.30, 4.60)) were significantly associated with ADHD (Table 5).

## 4. Discussions

The behavioral disturbance of children is the main hallmark that indicates children's understanding of the world. Behavioral disturbance was found to have a high implication for their social, academic, or occupational functioning. ADHD has a severe behavioral implication to the cognitive and social development among children. The persistent behavioral disturbance in children with ADHD may lead to major psychiatric disorders during adulthood.

The findings of the present study on the prevalence rate of ADHD are consistent with the findings of the studies done in Egypt, which was 6.9% [15]; in Kenya, which was 6.3% [18]; in the Democratic Republic of Congo, which was 6% [19]; in Spain, which was 6.8% [8], in the USA, which was 8.2%, [7], and in studies pooled worldwide, which was 7.2% [3], as well as in review studies which were 5.9%-7.1%. [4]. However, this finding was higher than the study done in Butajira, southern Ethiopia which was 1.5% [21] and Nigeria which was 3.2%. On the other hand, this finding was lower than in Venezuela, 10.03% [10]; in Saudi Arabia [11] and in Jeddah, 11.6% [12]; in Iran, 17%-25.8% [13]; in Nigeria, 23.15% [16]; and in Egypt, 19.7% [20]. The variation might be because of sociocultural differences, age demarcations, differences in sample size, and differences in the data collection tools used, like the use of some diagnosis criteria tools.

Gender was associated with ADHD, and males were almost two times more likely to have ADHD than females. This explanation agreed with several other studies [15, 16, 20]. This may be due the anatomical nature of maleness which is a risk for ADHD; a former study explained that the slightly larger heads of males might have put males more susceptible to pressure and head injury at birth [34]. The skeletal immaturity of boys relative to girls may cause boys to be more vulnerable to damage and predispose the development of ADHD later on among those children [35]. Gender differences might be due to referral bias as males are more likely to present with more externalizing symptoms (such as hyperactivity or impulsivity and physical aggression) than females. However, females are more likely to present with more internalizing symptoms (such as being withdrawn, being in a state of anxiety, and having low self-esteem) than males [36]. Children living with single parents are five times more likely to have ADHD than children living with both parents. This finding is in line with those of other studies showing that the separation of the child from one or both parents early in life is associated with ADHD [20, 37, 38]. Parental separation has negative effects on the child's behavior due to various factors such as inconsistent parenting, more punishment, violence, and criticism [38].

It was found that the first-born child was more than two times more likely to develop ADHD than the second and above child in the birth order in this study. This was similar with the study conducted in Egypt [37]. The reason why the risks for ADHD symptoms are greater among children who are first in the birth order might be due to poor mother-to-child attachment. The first-born child has a special position in some families and this may act as one of the risk factors for ADHD (e.g., overprotection and spoiling). The first child may also likely encounter some problems during pregnancy and labor such as the lack of prenatal care and narrow pelvis in primigravida that may lead to labor complications. This also agrees with reports that children who have a special position in the family and who receive overprotection and spoiling are more liable to develop ADHD than other children [39].

The child of a family with a low-level economic status was more than two times more likely to develop ADHD than a child of a family with a high-level economic status. This was in line with several previous studies [11, 20, 40]. Children who belong to a lower social class are at risk of having various psychiatric problems including ADHD. This may be because of pregnancy- or delivery-related complications (vaccination, prenatal follow-up, etc.) and malnutrition which are commonly associated with mothers with a poor socioeconomic status. This study showed no significant association between parents' education and developing ADHD in children, which was in line with some studies [11, 20]. However, it was in disagreement with a previous study [41] which has reported a high prevalence of ADHD in children from parents with a low level of education [42].

## 5. Limitations of the Study

The Disruptive Behavior Disorder Rating Scale is not validated in the Ethiopian setting. Recall bias may be present for childhood health status before six years old, child feeding style in the first six months of life, maternal health status during pregnancy, and complication at delivery.

## 6. Conclusion

The prevalence of ADHD in Ethiopia is similar to the prevalence of ADHD in most African countries and worldwide. ADHD is associated with sex, living with single parents, child birth order/rank, and low socioeconomic status of the family. Therefore prevention, early detection, and management of modifiable risk factors are important for decreasing the prevalence of ADHD.

## Figures and Tables

**Table 1:** Sociodemographic characteristics of children aged 6 to 17 years old.

Sociodemographic characteristics		Frequency	Percent (%)
Sex	Male	640	51.7
	Female	598	48.3
Age in years	6-12	894	72.2
	13-17	344	27.8
Ethnicity	Oromo	1208	97.6
	Others^∗∗^	30	2.4
Religion	Protestant	1003	81
	Orthodox	167	13.5
	Muslim	68	5.5
Child education	0-4th grade	1052	85
	5th-8th grade	186	15
Living circumstance	Living with both parents	1195	96.5
	Living with a single parent	43	3.5

^∗∗^Amhara, Wolaita, and Sidama.

**Table 2:** Sociodemographic characteristics of children's family.

Sociodemographic characteristics of the family (*n* = 1238)		Frequency	Percent (%)
Family marital status	Married	1202	97.1
	Divorced/widowed	36	2.9
Child's mother alive	Yes	1224	98.9
	No	14	1.1
Mother's educational level	Unable to read and write	716	58.5
	Primary school^1^	455	37.2
	Secondary and above^2^	53	4.3
Father's educational level	Unable to read and write	265	21.4
	Primary school^1^	678	54.8
	Secondary and above^2^	295	23.8
Child birth rank/order	First child	553	44.7
	Second child and above	685	55.3
Family size	>4 children	845	68.3
	1-4 children	393	31.7
Wealth Index	Lowest	416	33.6
	Middle	413	33.4
	Highest	409	33.0

^1^Grade one to grade eight. ^2^Grade nine and above.

**Table 3:** Clinical/biological factors of children and their mothers.

Clinical factors		Frequency	Percent (%)
Maternal health status during pregnancy	Sick	30	2.4
	Healthy	1208	97.6
Child health status before 6 years of age	Sick	26	2.1
	Healthy	1212	97.9
Maternal substance use during pregnancy	Alcohol/khat	45	3.6
	None	1193	96.4
Family history of mental illness	Yes	37	3
	No	1201	97
History of head trauma	Yes	22	1.8
	No	1216	98.2
Duration of pregnancy	Preterm	20	1.6
	Full term	1218	98.4
Complication at delivery	Yes	22	1.8
	No	1216	98.2
Child feeding style during the first six months	Bottle feeding	40	3.2
	Breast feeding	1198	96.8

**Table 4:** Prevalence of ADHD subtypes by sex.

Sex	ADHD subtypes					
	ADHD-IN		ADHD-HI		ADHD-C	
	Number	%	Number	%	Number	%
Male	34	59.6	17	77.2	8	72.7
Female	23	40.4	5	22.8	3	27.3
Total	57	100	22	100	11	100

**Table 5:** Associated factors with ADHD among children aged 6 to 17 years.

Explanatory variables	With ADHD		Without ADHD		Crude odds ratio (95% CI)	AOR (95% CI)
	Number	%	Number	%		
Sex						
Male	59	9.2	581	90.8	1.9 (1.18-2.91)	1.81 (1.13-2.91) ^∗^
Female	31	5.2	567	94.8	1	1
*P* value					0.007	0.014
Family marital status						
Divorced/widowed	12	33.3	24	66.7	7.21 (3.47-14.95)	0.8 (0.1-5.14)
Married	78	6.5	1124	93.5	1	1
*P* value					<0.001	0.8
Living circumstances						
Living with single parent	14	32.6	29	67.4	7.11 (3.6-14)	5.0 (2.35-10.65) ^∗^
Living with both parents	76	6.4	1119	93.6	1	1
*P* value					<0.001	<0.001
Child birth order/rank						
First child	20	3.6	533	96.4	3 (1.82-5.05)	2.4 (1.30-4.25) ^∗^
Second child and above	70	10.2	615	89.8	1	1
*P* value					<0.001	0.005
Family size						
>4 children	67	8.1	764	91.9	1.5 (0.9-2.4)	0.99 (0.5-1.8)
1-4 children	23	5.7	384	94.3		
*P* value					0.127	0.97
Wealth index						
Lowest	49	11.8	367	88.2	3.5 (1.9-6.4)	2.43 (1.28-4.58) ^∗^
Middle	26	6.3	387	93.7	1.8 (0.9-3.4)	1.4 (0.7-2.8)
Highest	15	3.7	394	96.3	1	1
*P* value					<0.001	0.006
Duration of pregnancy						
Preterm	4	20	16	80		
Full term	86	7.1	1132	92.9		
*P* value						
Complication at delivery						
Yes	5	22.7	17	77.3		
No	85	7	1131	93		
*P* value						
Child feeding style during						
The first six months of life						
Bottle feeding	5	12.5	35	87.5		
Breast feeding	85	7.1	1113	92.9		
*P* value						

^∗^Statistically significant (*P* value < 0.05); 1 = reference.

## Data Availability

The datasets used and/or analyzed during the current study are available from the corresponding author on reasonable request.

## Abbreviations

ADHD: Attention deficit hyperactivity disorder
ADHD-I: Attention deficit hyperactivity disorder inattentive
ADHD-HI: Attention deficit hyperactivity disorder hyperactive/impulsive
ADHD-C: Attention deficit hyperactivity disorder combined
AOR: Adjusted odds ratio
CI: Confidence interval
DSM-IV: Diagnostic and Statistical Manual of Mental Disorder Fourth Edition
ICD-10: 10th revision of the International Statistical Classification of Diseases and Related Health Problems.
